# On Apoplexy and Gout

**Published:** 1804-04-01

**Authors:** 


### *To the Editors of the Medical and Phyfical Journal.*

Gentlemen,

I Have been a constant Reader of your Journal since its publication, and have frequently been highly interested with many of the very useful papers it has contained; but I must confess to you that, within the last two years, its productions have appeared to me less valuable, and have tended more to embarrass than elucidate the Science of Medicine. Instead of liberal discussion on difficult points of pathology, which might lead to useful discovery of means that might alleviate or remove disease, it would seem that victory in controversy has veiled the sentiments of many of your correspondents, and led to an illiberal and ungentlemanly conduct.—What can be more gratifying than the plain and unadorned relation of facts, or opinions, which accompany the details of the well-informed and modest mind? I need not refer to verbal quotations to strengthen my assertion; your various numbers of late will singly supply abundant proof of its truth; but your last, in particular, contains a paper which shows such determined marks of hostility, and a determination to subdue, that I think none of your readers can approve the selfishness it betrays, or assent to the opinions which it unfolds.

If your correspondents would honestly detail to you the many many varieties of disease they meet with in their practice, if they would fairly state to you how inefficient, on many occasions, are the most powerful means, where frequently the most trifling remedies are followed with success, great advantages might accrue to society; and medicine, on most occasions, instead of being a complicated, would be rendered a machine of easy movement. Theories are of little value if they do not lead to successful practice; and the patient may be dead before this or that opinion shall decide on the remedy for his welfare. Of what worth can it be to me, or my surrounding friends, whether apoplexy depends on effusion, or a defective nervous energy, if the means adopted for its removal avail nothing, and one I highly esteem is sent to the grave? Ingenious men have no difficulty in forming theories to explain difficult appearances; but I would enquire, whether our knowledge is sufficiently distinct of the operations of the nervous system, and the relation between this portion of our frame, and the other parts of the system, to warrant us in deviating from the path of successful experience?

With respect to apoplexy, I have seen many cases where bleeding has quickly sent the patients to the grave. I have also seen emetics given with no immediate bad effect, but where the patient has afterwards languished in hopeless debility for years. I have also known where stimulants have been given, and where neither bleeding, nor vomiting, nor purging were employed, that the patient, who was in years, from not being able to speak distinctly, or move even a finger, has so far recovered as to walk round her premises, and converse with her friends. It has always appeared to me a necessary consideration, on being called to an apoplectic patient, that my views of the cure were not so much to be directed by the disease being apoplexy, as in directing my mode of cure to the removal of habits, or their consequences. I am fully persuaded if more attention was paid to this circumstance, more lives might be saved; and, though the lost energy of the system might never be restored to its former vigor, yet that we might so far recover its tone as to make life move on with ease, if if not with delight. From a considerable share of experience, I know well that the stomach will direct by its feelings the comfortable movements of the whole frame, and I am fully persuaded it has great influence in producing the apoplectic state, by uneasy irritations existing in it. I know well that tea for breakfast has in many led to feelings which have approached the apoplectic state. I know also that the drinking of rum has generated an acid in the stomach, which has produced an irritation there that has led to insensibility for a considerable time. I need only appeal to those who accustom themselves to take hot water with spirits at bed time, and make a breakfast the next morning of tea, to be informed how frequently they have languished before dinner, and how often they have sat down to a plentiful repast without being able to eat. Nor need I ask them how often their minds have been wavering, and discovered a temporary absence of thought, and a suspension of its other faculties? What is this but an approaching state of apoplexy, or a diminution of feeling? In such instances, I conceive, neither repletion, nor inanition, have any thing to do in producing apoplexy, but that irritations, excited in the stomach by peculiar means, are the sole cause in ninety-nine instances of a hundred.

I have something also to state to you on the gout, and I wish my experience could assure you of its removal by any means, if there be a want of resolution. Habit so completely subdues resolution, that I have met with very few, who after a time have not given way to inclination, and whatever good has been done, has very shortly been undone by reverting to former modes of living, and gout has returned with ten-fold violence. But I can relate the history of others, whose steady perseverance in plans laid down for their good, has amply rewarded their resolution, and though the gout had attacked them with the utmost violence, they continue to escape its painful and subduing efforts by their well judged prophylactic plan. Gout appears to me to be a general disease, and not to be cured by local means. In most gouty persons there is a highly irritable nervous system, whose balance with other parts of the body is destroyed, either by artificial means, or a defective structure. Where the latter prevails, little good is to be done; but in the former state of disease, every advantage may be derived from diet, and the employment of means which allay irritation through the medium of the skin. I know a gentleman, whose father died a martyr to the gout, who has himself been subject to its most violent attacks, who by resolution in adhering to a plan of diet recommended to him, has been almost entirely free from it for several years. This gentleman, from a belief that a bottle of wine after dinner, with ardent spirits and water, or punch after supper, would completely remove the gout, found by painful experience that he only increased its tendency tendency to return, and at length paralytic symptoms showed themselves. Since that time, which is now some years since, he has been exceedingly regular in his diet, and he enjoys better health than he had done for many years.

This gentleman is a striking instance of gout, as well as apoplexy, being connected with a peculiarly irritable state of the stomach. He can induce gout at any time by cucumbers, or a few grapes, or by eating any thing that has a tendency to produce acidity. I have attended another gentleman, who is so extremely irritable, and whose father was very gouty, who has had several attacks of it, from drinking a few glasses of wine, or brandy and water, and so strong is its tendency to recur, that the most trifling deviation from the most abstemious regimen, makes him feel its return. His persevering resolution in regimen is amply rewarded by good health and activity. Is it reasonable to suppose, that in either of these cases, with many others I could enumerate, that the local application of cold water would really cure the gout, if no attention was paid to the general habit of the patient? It appears to me so contrary to all sound experience, that medical men must wish for farther proof, before such a remedy can be generally employed with confidence, or employed at all without great hazard, if general means are not at the same time enjoined.

MEDICUS.

